# Anemia in Patients with Type 2 Diabetes Mellitus

**DOI:** 10.1155/2015/354737

**Published:** 2015-11-11

**Authors:** Jéssica Barbieri, Paula Caitano Fontela, Eliane Roseli Winkelmann, Carine Eloise Prestes Zimmermann, Yana Picinin Sandri, Emanelle Kerber Viera Mallet, Matias Nunes Frizzo

**Affiliations:** ^1^Regional University of Northwestern Rio Grande do Sul (UNIJUÍ), Ijuí, RS, Brazil; ^2^Program in Respiratory Sciences, the Federal University of Rio Grande do Sul (UFRGS), Porto Alegre, RS, Brazil; ^3^Department of Life Sciences, the Regional University of Northwestern Rio Grande do Sul (UNIJUÍ), Rua do Comércio No. 3000, Bairro Universitário, 98700 000 Ijuí, RS, Brazil; ^4^Program in Integral Attention to Health (PPGAIS-UNIJUI/UNICRUZ), Ijuí, RS, Brazil; ^5^Program in Pharmacology of the Health Sciences Center, The Federal University of Santa Maria (UFSM), RS, Brazil; ^6^Cenecista Institute for Higher Education, Rua Dr. João Augusto Rodrigues 471, 98801 015 Santo Ângelo, RS, Brazil

## Abstract

The objective of this study was to evaluate the prevalence of anemia in DM2 patients and its correlation with demographic and lifestyle and laboratory variables. This is a descriptive and analytical study of the type of case studies in the urban area of the Ijuí city, registered in programs of the Family Health Strategy, with a total sample of 146 patients with DM2. A semistructured questionnaire with sociodemographic and clinical variables and performed biochemical test was applied. Of the DM2 patients studied, 50 patients had anemia, and it was found that the body mass items and hypertension and hematological variables are significantly associated with anemia of chronic disease. So, the prevalence of anemia is high in patients with DM2. The set of observed changes characterizes the anemia of chronic disease, which affects quality of life of diabetic patients and is associated with disease progression, development, and comorbidities that contribute significantly to increasing the risk of cardiovascular diseases.

## 1. Introduction

Diabetes mellitus (DM) is a metabolic disorder of great impact worldwide. Epidemiological data showed that in 2010 there were 285 million people affected with the disease in the world, and it is estimated that in the year of 2030 we will have about 440 million diabetics [1]. Its worldwide prevalence is increasing fast among developing countries. The type 2 diabetes affects about 7% of the population [2].

The increasing prevalence of type 2 diabetes mellitus (DM2) has become a major public health concern. The diabetic patients' number has been increasing due to population and urbanization growth, increase in the prevalence of obesity and sedentary lifestyle, and the longer survival of patients with DM [3]. Diabetes is a highly disabling disease, which can cause blindness, amputations, kidney disease, anemia, and cardiovascular and brain complications, among others, impairing the functional capacity and autonomy and individual quality of life [4].

The disease can be classified into two predominant types, as type 1 DM (DM1), defined by the destruction of pancreatic *β*-cells and the absence of endogenous insulin, and as DM2, insulin resistance characterized by a frame, generally associated with obesity. Both types are featured by hyperglycemia above. Insulin resistance reduces glucose tolerance especially in muscle cells and adipocytes, where glucose uptake is insulin dependent. This causes glucose accumulation in the circulation and consequently a hyperglycemic state, generating homeostatic and systemic imbalance [5].

Diabetes is considered a major cause of premature death, because of the increased risk for developing cardiovascular diseases, which contribute to 50% to 80% of patients deaths due to increased levels of serum cholesterol and triglycerides. Cardiovascular diseases include diseases of the circulatory system, comprising a wide range of clinical syndromes, the main cause of atherosclerosis, which also increases the risk of acute coronary syndromes. The incidence of cardiovascular diseases reaches 20% in diabetics after a period of about 7 years [6].

Hyperglycemia has a direct relationship with the development of an inflammatory condition showed by the increased expression of proinflammatory cytokines such as IL-6, TNF-*α*, and NF*κ*B. Thus, diabetes, as well as hyperglycemia due to its nature, is also an inflammatory disease character. Studies show that the longer the duration of the disease and/or loss of glycemic control, the higher the inflammatory process [7, 8].

The elevation of proinflammatory cytokines plays an essential role in insulin resistance and induces the appearance of cardiovascular complications diabetic micro- and macrovascular, kidney disease and anemia. By increasing especially IL-6, antierythropoietic effect occurs, since this cytokine changes the sensitivity of progenitors to erythropoietin (erythroid growth factor) and also promotes apoptosis of immature erythrocytes causing a decrease, further, in the number of circulating erythrocytes and consequently causing a reduction of circulating hemoglobin [7, 9].

It should also be noted that, due to the development of diabetes mellitus, the nephropathy may arise, which further undermines the renal production of erythropoietin, positively contributing to an increased anemic framework [9, 10]. According to Escorcio et al. [11] approximately 40% of diabetic patients are affected by kidney diseases. The decreased renal function and proinflammatory cytokines are the most important factors in determining reduction of hemoglobin levels in those patients. The inflammatory situation created by kidney disease also interferes with intestinal iron absorption and mobilization of inventories [12]. Therefore, diabetic patients with kidney disease have the highest risk for developing anemia [11].

The National Kidney Foundation defines anemia in chronic kidney disease as Hb level < 13,5 g/dL in men and 12,0 g/dL in women [13]. Anemia represents an emerging global health problem that negatively impacts quality of life and requires an ever-greater allocation of healthcare resources [14]. The anemic framework promotes reduced exercise capacity, fatigue, anorexia, depression, cognitive dysfunction, decreased libido, and other factors, which increase cardiac risk patients and depress the quality and life expectancy of the same [15]. Under these circumstances, anemia in patients with diabetes must be treated once diagnosed, since it may contribute to the pathogenesis and progression of cardiovascular disease and serious diabetic nephropathy and retinopathy. The regular screening for anemia, along with other complications associated with diabetes, can help slow the progression of vascular complications in these patients [16].

Anemia in diabetic person has a significant adverse effect on quality of life and is associated with disease progression and the development of comorbidities [7], as obesity and dyslipidemia that are strongly associated with diabetic framework and significantly contribute to increasing the risk of cardiovascular diseases [8]. Thus, the present study is to evaluate the prevalence of anemia in a sample of patients with type 2 diabetes who are living in a city in the northwest of the Rio Grande do Sul state, registered in the Family Health Strategies, and check its correlation with demographic, lifestyle, and laboratory variables of patients.

## 2. Methods

This is a descriptive and analytical study of the type of case studies in patients with DM2 and ages less than 75 years living in the urban area in the city of Ijuí RS, registered in programs of the Family Health Strategy (FHS) in this city. The study was conducted from January 2010 to January 2013, after agreement by the Research Ethics Committee of the Regional University of Rio Grande do Sul State Northwest (UNIJUÍ) (Opinion number 091/2010). All participants signed the informed consent in this research.

The sample size was calculated by StatCalc application EpiInfo 3.5.3, considering the prevalence of nonspecific outcome of 50%, 5% error, and 95% level of reliability, which resulted in a sample of 269 patients. Foreseeing possible losings a percentage of 5% of this number was added, a total sample of 283 patients with DM2.

The study excluded those patients who had difficulties to understand the proposed procedures, those who were bedridden, and those who had difficulty walking.

The invitation to participate in the study was made to patients during home visits, with the monitoring of community health workers when possible. At the moment of visit, the research objectives were explained for the patient and the dates of the interviews were fixed with those who agreed to participate, in addition to scheduling the clinical and laboratory reviews, held, respectively, in Physiotherapy Clinic and the Laboratory of Clinical Analysis of UNIJUÍ (UNILAB).

The interviews and tests were conducted by trained health professionals. Data collection was performed by applying a semistructured instrument. The presence of anemia was considered as the dependent variable; the patient was considered anemic, according to the World Health Organization reference values [17]. Thus, the patient was considered anemic patient when the blood count hemoglobin < 12 g/dL and <14 g/dL for females and males, respectively. The independent variables analyzed were as follows:

- Sociodemographic characteristics:
  - age (in years);
  - sex (female/male).
- Health condition:
  - time of diagnosis of type 2 diabetes (in years);
  - advanced age (over 60 years).
- Comorbidities:
  - presence of hypertension (yes/no);
  - cardiac and/or respiratory (yes or no), analyzed according to the patient's report when asked about the presence of these diseases;
  - dyslipidemia (yes/no), diagnosed by biochemical tests;
  - obesity (yes/no), when the value of body mass index was ≥30,0 kg/m^2^ for patients up to 59 years old and ≥27,0 kg/m^2^ for patients aged 60–75 [18].
- Lifestyle:
  - smoking (yes/no);
  - alcohol consumption (yes/no);
  - physical inactivity (yes/no);
  - stress (yes/no).
- Eating habits, investigated through questioning a high salt diet (yes/no).

Every patient who declared himself a smoker at the moment of evaluation is considered smoker, regardless of the amount of cigarettes consumed; and alcoholic is the person who reported excessive consumption of alcohol during the study period, at any frequency. Excessive salt intake was measured by the question: Do you put much salt in your food? Stress was assessed by the question: Do you consider yourself a stressed person? There were classified physically inactive patients who reported not performing any type of regular exercise with the lowest frequency of three times a week.

The evaluation of anthropometric data, including the measurement of body weight (in kilograms) on digital scale, was performed (Toledo); height (in meters) in stadiometer (Toledo) and waist circumference (WC) were measured at the midpoint between the last rib and the iliac crest using flexible standard tape and nonextensible, defining measure of 0,1 cm, according to techniques recommended [19]. The body mass index (BMI) was calculated by dividing body weight and the square of height: kg/m^2^.

At the end of the clinical evaluation, an appointment was made with the date and the time of collection of blood from each patient. Patients personally received clarification on the procedures of collection and were instructed to fast for at least eight hours prior to the blood collection, in addition to writing instructions and containers for the collection of the first urine in the morning. Among the laboratory tests that were performed are the creatinine dosage and blood glucose by enzymatic Trinder method [20]. In addition, the collection and enforcement of the blood count were performed to evaluate the presence of hematological disorders in patients with DM2. The blood sample, was also used the serum of patients after venipuncture and centrifugation of whole blood for biochemical measurements, as well as whole blood anticoagulant containing standard for hematologic examinations.

The patient who presented two or more of the following criteria proposed by the National Cholesterol Education Program was classified as having metabolic syndrome: [21] increased waist circumference (>88 cm women and >102 cm men); elevated serum triglycerides (≥150 mg/dL) or decreased HDL cholesterol (<40 mg/dL men and <50 mg/dL women); and hypertension (diagnosed or identified through the use of antihypertensive medication).

Renal function was assessed by the value of serum creatinine, obtained by biochemical tests. The glomerular filtration rate is estimated by the Cockcroft-Gault calculated using the formula available on the websites of the Brazilian Society of Nephrology (SBN) of the National Kidney Foundation [22]. We considered impaired renal function the values above 1,2 mg/dL in the serum creatinine [23] and the GFR less than 60 mL/min/1,73 m^2^ estimated by the Cockroft-Gault equation [24] representing a decrease of about 50% of normal renal function and, below this level, increasing the prevalence of complications of chronic kidney disease [25]. For the use of the Cockcroft-Gault equation, the ideal weight of the patient was computed using the Lorenz formula, which puts the ideal body weight for the subjects height in cm function [26].

For processing the data, we used the Statistical Package for Social Science (SPSS) (version 18.0, one Chicago, IL, USA). In the statistical analysis, all variables were tested for normality using the Kolmogorov-Smirnov (KS) test. The qualitative variables are presented as frequencies and percentages and quantitative variables as average and standard deviation (average ± SD) ormedian (minimumand maximum). Mann-Whitney tests were used to compare two independent groups with abnormal distribution, Student's *t*-test was used for normally distributed variables, and the Chi-square test and Fisher's exact Pearson were used to compare categorical variables in order to verify differences of variables between patients with and without anemia. The Spearman correlation coefficient was used to evaluate the correlation between clinical and biochemical parameters with the hemoglobin level. *p* < 0,05 was considered statistically significant. All tests were applied with a Confidence Interval (CI) of 95%.

## 3. Results

283 patients with DM2 were suitable to the study inclusion criteria and were selected for home visits and invitation to participate in the study, according to data collected from health professionals in FHS or the medical records of the patients belonging to nine FHS in the city of Ijuí, RS. Of these, 64 patients were not included in the study for the following reasons: contact absence; refusal to participate; and not identifying the address informed and 73 individuals were not included due to insufficient data to evaluate the hematologic changes, since they did not undergo blood tests for hemoglobin count, a total sample of 146 type 2 diabetic patients in this study, of which 50 had anemia, corresponding to 34,2%.

The study population had an average age of 60,9 ± 8,9 years, body mass index of 31,2 ± 5,8 kg/m^2^, and a median of disease diagnosis time of 5,0 years (0,5–40,0 years).

We analyzed the dependent variable “anemia” according to some characteristics of patients with DM2. For time of diagnosis of the disease, old age, metabolic syndrome, renal dysfunction by creatinine, and the Cockcroft-Gault equation, there was no difference between the presence and absence of anemia (*p* > 0,05). However, variables, body mass, and hypertension showed significance to the outcome studied (*p* < 0,05). These data are presented in Table 1.

We observed statistically significant difference in hematological variables between groups with and without anemia (*p* < 0,05). The same is observed with respect to glucose, however, with higher values in the group without anemia. There was no statistically significant difference in creatinine variable (*p* > 0,05). These data are presented in Table 2.

Table 3 shows the correlations between the clinical and biochemical parameters with hemoglobin. It is observed that there are positive and weak correlation between glucose and hemoglobin and negative and weak correlation between BMI and hemoglobin.

## 4. Discussion

Often, chronic diseases, such as DM, are accompanied by mild-to-moderate anemia, often called anemia of inflammation or infection or anemia of chronic disease [27]. Andrews and Arredondo [28] determined the presence of anemia in type 2 diabetic patients as well as evaluating the expression of genes related to inflammation and immune response. The results found by the authors demonstrate that diabetic patients with anemia exhibit increased expression of proinflammatory cytokines as compared to diabetic patients only. In anemic patient increase in IL-6 production, as well as B cell activity, was confirmed which reinforces the association between IL-6 and antierythropoietic action. Moreover, the diabetic and anemic patients had high levels of C-reactive protein and ferritin ultrasensible; however, these diabetic and anemic patients had low iron contents, showing that ferritin increases were associated with chronic inflammatory process present in diabetes [28].

In this study, there was a higher prevalence of obesity and higher mean BMI and waist circumference in anemic patients when compared to nonanemic ones; however, there was a statistically significant difference between the groups only for body mass variable. Anemia in diabetic patients is also related to obesity, BMI, and high waist circumference. The obesity or accumulation of circulating fatty acids is associated with the development of an inflammatory state that predisposes the development of insulin resistance. Insulin resistance reduces glucose tolerance especially in adipocytes and muscle cells, in which glucose uptake is insulin. This causes glucose accumulation in the circulation and consequently a hyperglycemic state [29].

Adipose tissue has more recently been recognized as a metabolically active organ system linking the endocrine and immune systems; furthermore it is the source of a variety of cytokines. Higher baseline BMI remained a predictor of additional adjustments for blood pressure level and the presence or absence of diabetes mellitus. Similar to TNF-alpha, IL-6 is a proinflammatory adipokine that correlates with body weight and insulin resistance [30].

The increased inflammatory activity in adipose tissue of obese patients favors the production of hepcidin that in anemia of chronic disease is increased during infection and inflammation, causing a decrease in serum iron level through a mechanism that limits the availability of iron. The association of higher iron stores with diabetes and insulin resistance has been repeatedly confirmed by many investigators. Ferritin levels were found to predict a higher rate of diabetes in prospective studies and case-control cohorts. Furthermore, serum ferritin was positively associated with body mass index (BMI), visceral fat mass, serum glucose levels, insulin sensitivity, and cholesterol levels [31–33].

In addition, it was found in this study that the prevalence of hypertension in diabetic patients that were anemic was significantly higher when compared to nonanemic ones. This association is of concern considering that hypertension in diabetic increases the risk of cardiovascular complications such as heart failure, stroke, tissue inflammation, and atherosclerosis [4].

According to Ximenes et al. [34] anemia is a prevalent comorbidity in patients with hypertension and when present, patients have more severe symptoms and worse functional capacity as well as increased mortality. The knowledge that anemia worsens the symptoms of hypertension is not new, but, in recent years, the magnitude of the anemia associated with this disease has become more evident. The main causes that contribute to anemia in patients with hypertension are nutritional deficiencies especially iron deficiency and chronic inflammation [34].

It was observed in the present study that there are decreased values of hemoglobin, hematocrit, and red blood cells in anemic patients, which can be associated with a normocytic normochromic anemia, characteristic of an anemia of chronic disease (ACD). ACD is a light-to-moderate anemia shortening the survival of red blood cells (about 80 days instead of 120 days normal). This phenomenon is attributed to hyperactivity state mononuclear phagocyte system, triggered by infectious, inflammatory, or neoplastic process, leading to early removal of circulating red blood cells. Inadequate bone marrow response observed is due basically to inappropriately low Secretion of Erythropoietin (EPO), decreased bone marrow response to EPO, and decreased erythropoiesis consequent to lower supply of iron to the bone marrow [35].

One explanation for this bone marrow response is directly related to the activation of macrophages and the release of inflammatory cytokines, particularly IL-1, IL-6, tumor necrosis factor (TNF a), and interferon gamma (INF g) which act by inhibiting the proliferation of erythroid precursors and therefore inhibit erythropoiesis. Furthermore, the suppressive action of these cytokines on erythropoiesis stimulating overcomes the action of EPO resulting in decreased bone marrow response to EPO and erythropoiesis [36].

Also it should be noted that there was no hemoglobin correlation with creatinine or statistical differences in creatinine values and glomerular filtration rate estimated between groups, indicating once again that anemia by chronic disease was inflammation triggered and the reduction renal function affects the production of EPO.

The limitations in this study refer to the fact that the assessment of glycemic control in diabetic patients was performed by means of fasting glucose that is a momentary biochemical analysis, does not represent the average glucose of patients, and may also occur interfering in the examination, as the effect of hypoglycemic agents, promoting a reduction in glucose levels. In this sense, the gold standard for assessing glycemic control would be the achievement of HbA1c (glycated hemoglobin), which is one of the most important tools to assess glycemic control of patients with diabetes, as they express the average amount of glucose in the last three months, and this can infer the diabetes control efficiency and suggest the need for adjustments.

Therefore, it is suggested that further studies should be conducted using test glycated hemoglobin, which currently is already considered an essential parameter in the DM control evaluation, in order to relate hyperglycemia, inflammation, and anemia.

## 5. Conclusion

Patients with DM2 and anemia were those with high body mass, hypertension, increased waist circumference, and longer time of the disease. This set of changes characterizes the anemia as chronic disease, which has a significant adverse effect on quality of life of diabetic patients and is associated with the progression of the disease; the development of comorbidities significantly contributes to the increased risk of cardiovascular disease. However, against what was expected, the results of blood glucose were higher in nonanemic patients, which is contradictory due to the anemia of these patients being associated with an inflammatory condition, for being characterized as normocytic normochromic anemia. Deepening the study of the issues raised throughout this work provides knowledge for the establishment of new strategies for glycemic control, which can increase the research and correlate some analytical parameters, such as HbA1c, I1-6, VHS, and PCR.

## Figures and Tables

**Table 1:** Characteristics of patients with diabetes mellitus type 2 according to the presence of anemia.

Variables	Anemia		
	Yes (*n* = 50)	No (*n* = 96)	*p* value
Gender			0,059^*£*^
Male	23 (46,0)	29 (30,2)	
Female	27 (54,0)	67 (69,8)	
Age (in years)	61,8 ± 9,5	60,5 ± 8,7	0,274^*µ*^
Body mass (kg)	83,6 ± 16,8	77,5 ± 13,5	0,019^*¥∗*^
Height (m)	1,61 ± 0,08	1,59 ± 0,09	0,287^*¥*^
BMI (kg/m^2^)	32,2 ± 6,0	30,6 ± 5,7	0,126^*¥*^
Waist circumference (cm)	105,9 ± 15,9	104,7 ± 11,5	0,626^*¥*^
Time of diagnosis of DM2 (in years)	6 (0,5–40,0)	5 (0,6–40,0)	0,148^*µ*^
Advanced age	30 (60,0)	55 (57,3)	0,753^*£*^
Hypertension	42 (84,0)	65 (67,7)	0,035^*£∗*^
Dyslipidemia	23 (46,0)	48 (50,0)	0,646^*£*^
Obesity	38 (76,0)	65 (67,7)	0,297^*£*^
Metabolic syndrome	35 (70,0)	56 (58,3)	0,167^*£*^
Heart disease	10 (20,0)	18 (18,8)	0,856^*£*^
Respiratory disease	6 (12,0)	15 (15,6)	0,627^*€*^
Smoking	8 (18,0)	14 (14,6)	0,179^*£*^
Alcoholism	4 (8,0)	6 (6,3)	0,924^*€*^
Physical inactivity	23 (46,0)	50 (52,1)	0,485^*£*^
Stress	23 (46,0)	53 (55,2)	0,291^*£*^
Hypersodic diet	6 (12,0)	19 (19,8)	0,259^*€*^
Alteration of renal function by creatinine	9 (18,0)	18 (18,8)	0,912^*£*^
Alteration of renal function by Cockcroft-Gault equation	12 (24,0)	25 (26,0)	0,788^*£*^

DM2: diabetes mellitus type 2; ¥ indicates *p* value according to Student's *t*-test; *µ* indicates *p* value according to test of Mann-Whitney; *£* indicates *p* value according to test of Chi-square of Pearson; *€* indicates *p* value according to the exact test of Fischer; results presented on average ± standard deviation or median (minimal and maximal value) and number (percentage); *∗* was considered statistically significant.

**Table 2:** Biochemical and hematological variables in patients with DM2 according to the presence of anemia.

Variables	Anemia		
	Yes (*n* = 50)	No (*n* = 96)	*p* value
Hemoglobin (g/dL)	11,68 ± 0,81	13,32 ± 0,85	<0,0001^*¥∗*^
Hematocrit (%)	35,08 ± 5,23	40,45 ± 2,88	<0,0001^*µ∗*^
Red cells (millions/mm^3^)	4,23 ± 0,37	4,68 ± 0,34	<0,0001^*¥∗*^
Glycemia (mg/dL)	109,4 ± 40,5	133,6 ± 55,2	0,005^*µ∗*^
Creatinine (mg/dL)	1,05 ± 0,39	1,03 ± 0,27	0,944^*µ*^
Glomerular filtration rate (mL/min)	91,7 ± 41,9	79,9 ± 27,2	0,277^*∗∗*^

¥ indicates *p* value according to Student's *t*-test; *µ* indicates *p* value according to test of Mann-Whitney; *∗* was considered statistically significant; results presented in average ± standard deviation or median (minimal and maximal value). *∗∗* indicates mean glomerular filtration rate by Cockcroft-Gault equation.

**Table 3:** Coefficients of correlation between clinical and biochemical parameters with the hemoglobin in patients with diabetes mellitus type 2.

Variables	Hemoglobin	
	*p*	*R*
Age (in years)	0,492	−0,057
BMI (kg/m^2^)	0,051	−0,155
Time of diagnosis of DM2	0,466	−0,061
Glycemia (mg/dL)	0,004^*∗*^	0,235
Creatinine (mg/dL)	0,209	0,105
Glomerular filtration rate by the Cockcroft-Gault equation (mL/min./1,73 m^2^)	0,526	0,053

Spearman rank correlation test; *∗* was considered statistically significant.
